# Can Kindergarten Meals Improve the Daily Intake of Vegetables, Whole Grains, and Nuts among Preschool Children? A Randomized Controlled Evaluation

**DOI:** 10.3390/nu15184088

**Published:** 2023-09-21

**Authors:** Maja Berlic, Tadej Battelino, Mojca Korošec

**Affiliations:** 1 Department of Food Science and Technology, Biotechnical Faculty, University of Ljubljana, Jamnikarjeva ulica 101, 1000 Ljubljana, Slovenia; berlic.maja@gmail.com; 2 Preschool Galjevica, Galjevica 35, 1000 Ljubljana, Slovenia; 3 Division of Paediatrics, University Medical Centre Ljubljana, Bohoričeva ulica 20, 1000 Ljubljana, Slovenia; tadej.battelino@kclj.si; 4 Faculty of Medicine, University of Ljubljana, Vrazov trg 2, 1000 Ljubljana, Slovenia

**Keywords:** child nutrition, intervention, dietary record, food groups, food intake, recommendations

## Abstract

Surveys have indicated that preschool children do not consume adequate amounts of vegetables, fruits, whole grains, and nuts. This cross-sectional intervention study aimed to investigate whether a meticulously crafted meal plan for children of kindergarten age (5–6 years) could effectively enhance their daily intake of nutritious foods. Ninety-four healthy children from six kindergartens were enrolled in the study and were randomly assigned to a prototype group (PG) and a control group (CG). The PG kindergartens (*n* = 4) received a prototype 5-day meal plan that included regulated portions of vegetables, fruits, whole grains, and nuts adhering to dietary guidelines. Conversely, the CG kindergartens (*n* = 2) adhered to their standard meal plan. Participants maintained their usual eating habits outside of kindergarten and during weekends. Using the dietary assessment tool Open Platform for Clinical Nutrition (OPEN), combined with a 7-day dietary record of food consumed inside and outside the kindergarten, the average daily intake of specific food groups was assessed and compared with the Dietary Guidelines for Children. A total of 57 participants completed the study, 40 from the PG and 17 from the CG. Among the PG participants, the average daily intake of vegetables, whole grains, and nuts compared with the guideline recommendations was significantly higher than in the CG. Notably, only meals consumed within the kindergarten setting significantly improved the overall intake, with the outside intake having no significant effect. This study underscores the vital role of a well-designed and precisely executed meal plan in kindergartens in improving children’s intake of healthy foods. The findings could help drive positive changes in child nutrition within educational environments.

## 1. Introduction

The preschool years are a time of intense growth and development and a critical period in the establishment of lifelong eating habits [1]. Although the home environment is a key factor in shaping eating habits [2], more than 80% of preschool-aged children in high-income countries are enrolled in pre-primary education [3,4], where they may spend up to 251 days per year and as many as 9 h each day [4]. During this time, they are provided with at least three meals (breakfast, lunch, and snack), often accompanied by an additional snack. These meals are expected to meet more than 60% of children’s daily nutritional and energy needs. This high figure underscores the potential influence of kindergarten meals on children’s overall health, either to their advantage or disadvantage.

The interrelationship between nutrients, foods, and dietary patterns has important implications, particularly for preventing and developing NCDs such as cardiovascular disease, cancer, and diabetes [5,6]. According to the most recent Global Burden of Disease study, one out of every five premature deaths globally can be attributed to unhealthy diets [7]. Alarming trends of unhealthy dietary habits among children worldwide also play a role in the surging rates of obesity [1,8]. Adequate consumption of vegetables, fruits, whole grains, and nuts has been linked with reduced overweight and obesity in children [9]. Additionally, the consumption of these foods demonstrates an inverse correlation with body mass index [10]. Consequently, adopting kindergarten-based measures focused on promoting healthy food intake in children provides an opportunity to alleviate the burden of NCDs later in life [1].

Over the past decade, numerous European countries have undertaken various measures to enhance the nutritional environment within educational institutions [11]. However, the recent report of the European Food Safety Authority (EFSA) Comprehensive European Food Consumption Database shows that European children consume low amounts of vegetables and nuts, while fruit intake is slightly better but still too low [12].

In Slovenia, nutrition in kindergartens has been well established since 2005 when the Guidelines for healthy eating in educational institutions were accepted (hereafter “Guidelines”) [13]. Furthermore, the National School Nutrition Act [14] ensures that all children can access high-quality food regardless of socioeconomic status. Most kindergartens in Slovenia are municipal (public) and provide children with three to four daily meals, including breakfast, lunch, and one to two snacks. While these Guidelines are legally binding and firmly established, they allow for specific simplifications that manifest in insufficient vegetables, whole grains, and nuts, as highlighted in a report on professional nutrition monitoring in educational institutions [15].

Despite the large proportion of meals consumed by children in kindergarten, a separate Slovenian survey revealed that only 30% of young children eat vegetables daily, while 60% consume fruits. Additionally, 59% of Slovenian toddlers do not eat nuts [16]. The limited presence of whole grains, fruits, and vegetables in kindergarten diets is similarly reported in other studies worldwide [17,18,19].

Given the beneficial effects of consuming vegetables, whole grains, and nuts on health [20,21,22], coupled with the harmful effects of insufficient consumption or poor dietary habits and its associated economic burden [23], it is rational to explore strategies to improve the consumption of these foods in educational settings as well. While other studies have already described the contribution of preschool nutrition to the daily intake of food groups in children [18,19,24,25], we hypothesize that a specific prototype kindergarten meal plan that provides a higher (adequate) daily intake of vegetables, whole grains, and nuts based on the recommended daily food intake (RDFI) [13] will improve the intake of healthy foods in this age group.

Therefore, the main objective of this study was to investigate whether a carefully designed kindergarten meal plan can effectively improve the daily intake of healthy foods, especially vegetables, whole grains, and nuts, among children aged 5 to 6 years. This study had three endpoints: Firstly, to estimate the average food intake from seven food groups (fruits, vegetables, whole grains, refined grains and potatoes, nuts, meat and substitutes, and milk and milk products) in the group following a comparison prototype meal plan (prototype group: PG) with the usual meal plan (control group: CG). Secondly, to compare the contribution of the intervention eating plan (PG) to the daily intake of the seven food groups with that of the regular eating plan (CG). Finally, to compare the dietary intake between the participants of the two study groups with the RDFI.

## 2. Materials and Methods

### 2.1. Study Design and Settings

This 7-day cross-sectional intervention was designed in spring 2019, and participants were recruited in August and September 2019. The intervention study was conducted between March and June 2020.

Conceptually, a kindergarten menu designed in accordance with the Guidelines can be viewed as an array of versatile foods combined in kindergarten meals to provide optimal amounts of nutrients for children’s healthy growth and development. Nutrient amounts are proportional to children’s nutrient needs and the amount of time they spend in kindergarten. Because the Guidelines provide instructions on food groups and their frequency in the daily or weekly menus, the selection of foods varies among kindergartens and depends on the person designing the menus.

Twelve kindergartens situated within urban and suburban areas of Central Slovenia were contacted to participate in this study. Each kindergarten management team was briefed on the details and obligations of the study protocol. Inclusion criteria included written consent to participate and a commitment to fulfil all obligations without requesting material compensation. Based on the obtained consent from the kindergarten management teams before the study, six kindergartens were chosen—three from the Zasavje region and three from the Ljubljana region. The choice was made to ensure that all the kindergartens were from similar urbanized areas to avoid possible meal preparation and serving disparities. Recruitment of participants took place during the initial kindergarten meetings with parents when the study objectives, participation expectations, and the importance of informed parental consent were communicated. Approximately 20 children from each kindergarten were invited to participate to ensure a balanced representation of children from all kindergartens, as participants were burdened with completing a 7-day dietary record and received no financial compensation.

Participating kindergartens were randomly divided into two groups: PG, which included two kindergartens from each region (*n* = 4), and CG, which included one kindergarten from each region (*n* = 2). Randomization was performed in Excel using the RANDBETWEEN function to select the control kindergarten in each region. The VLOOKUP function was used to determine the name of the randomized kindergarten. Setting the targeted statistical power of the analysis at 90% with a two-sided test alpha (α) value of 0.05 and assuming a dropout rate of 10% and a sample size ratio between CG and PG of 0.5, the required number of participants per group to detect a difference of 60 g in daily vegetable consumption was 26 in PG and 13 in CG.

The PG was given a 5-day prototype (intervention) meal plan (Thursday to Wednesday), excluding weekends (Supplementary Table S1). In contrast, kindergartens in the CG used meal plans from the same period of the previous year to avoid bias caused by kindergartens knowing about their participation in the study (Tables S2 and S3). The participants’ diet outside of kindergarten did not change during the trial.

All meal plans were designed following specific Guidelines [13], with the difference being that the prototype meal plan meticulously followed the detailed recommendations, whereas the control meal plans followed only the general recommendations [13].

The daily meal plan comprised a list of foods to be used for each meal. The daily meals included the same quantities (g) of all individual foods and dishes as specified in the meal plan in the quantities (g) representing the portions served to each child. The approved costs for purchasing food were not exceeded in any participating kindergartens.

### 2.2. Participants

Inclusion criteria were as follows: a healthy child, aged 5–6 years, attendance at all kindergarten meals (breakfast, lunch, two snacks), and provision of a fully recorded consecutive 7-day dietary record. Gender was not an important factor. The exclusion criteria were all food allergies, chronic disease, and the need for dietary supplements.

### 2.3. Anthropometric Measurements

Data were obtained on the body weight and height of all participants to avoid bias due to a possibly higher food requirement. These were collected by a family pediatrician, who performed a regular medical examination before school enrollment between March and June 2020. Height was measured (±0.1 cm) using a stadiometer, and body weight (±0.1 kg) using an electronic scale. Participants wore light underwear and no shoes. The body mass index (BMI) was also recorded, and each child’s weight status was defined according to the International Working Group on Obesity‘s sex- and age-specific cut-offs for children [26].

### 2.4. Ethical Considerations

This study is part of a larger research project registered at ClinicalTrials.gov (NCT04252105) focusing on the impact of an antioxidant-rich kindergarten diet on oxidative stress in healthy children. It was conducted following the principles of the Declaration of Helsinki concerning ethical principles for medical research involving human subjects. Ethical approval was granted by the Medical Ethics Committee of the Republic of Slovenia (No. 0120-66/2019/8). Participation in this study was entirely voluntary, and the parents or guardians of all participants provided their informed consent prior to participation.

### 2.5. Dietary Survey

Kindergarten 5-day dietary record: All meals were consumed in the classrooms with typically 24 children and 1–2 teachers. Kindergarten teachers received detailed verbal and written instructions from a trained dietitian on completing the dietary record during the study. The design of dietary records adhered to EFSA-recommended guidelines [27]. Each teacher was provided with printed, 5-day close-ended forms containing all the foods specified in the meal plan. Additionally, a balance was supplied to measure the amount of food served and the amount of leftovers. Before the official start of the study, a trial recording was conducted to eliminate potential issues. Five consecutive daily meals were observed in twelve classrooms in six kindergartens. Teachers only recorded the beverages consumed as part of the meal. Water and unsweetened tea offered outside of meals were unequally consumed by participants and were therefore not included in the data. Importantly, no constraints or obligations were imposed on their food intake.

Seven-day dietary record of consumption outside of kindergartens: Parents were instructed to record their child’s dietary intake on the same days as kindergarten teachers and during weekends, employing either weighing or the estimated food intake technique. To facilitate this, they received printed, open-ended daily forms for seven consecutive days. These forms were structured following the standard meal plan: breakfast, snack, lunch, snack, and dinner, with provision for recording additional supplementary snacks. The forms also required details such as product (brand) names and ingredient quantities in grams. Detailed written instructions were also provided. In addition, parents received a booklet featuring household measures designed to aid in determining food intake (https://nijz.si/publikacije/slikovno-gradivo-s-prikazom-velikosti-porcij/ (accessed on 10 February 2020)). The booklet, created in alignment with the Pilot study for Assessment of Nutrient intake and food Consumption Among Kids in Europe (PANCAKE) [28] and previously utilized in the EU Menu Study, comprises 46 distinct images depicting various foods and simple recipes. Each image shows six different portion sizes used to evaluate the food pictured or foods of similar density, size, and shape. Parents were encouraged to be as accurate as possible and promptly record the quantities of food and beverages consumed.

### 2.6. Dietary Intake Data Processing

Assessment of food groups offered in the meal plans/meals: All meals were cooked and prepared in the kitchens of the kindergartens. Also provided were detailed recipes (*n* = 129), the quantities of foods used from all six kindergartens, estimated portion sizes, and the brand names of food products and beverages used to prepare the daily meals. All data were entered into the national reference database and dietary assessment tool Open Platform for Clinical Nutrition (OPEN) [29], where the dishes were classified into seven food groups: (1) meat and substitutes, (2) milk and dairy products, (3) fruits, (4) vegetables, (5) nuts, (6) whole grains, (7) refined foods including potatoes. The classification of starchy foods was performed manually. Foods containing cereals were classified as whole grain if they contained at least 30 g of whole grain per 100 g of product and more whole grain than refined grain [30]. Given that potatoes are a starchy food, they were classified together with refined grains due to their low dietary fiber content (≤6 g) [31]. Legumes were classified as vegetables [13]. Nuts were classified as a distinct category due to their low intake by children [16] and their beneficial effects on health [22]. The classification of mixed dishes is shown in Table S4.

The OPEN platform combines data on nutrient content in food obtained through chemical analysis with data from other reference databases. All values in the OPEN are expressed in g or mg per 100 g of edible parts of the food. Total fat (g) includes compounds determined by sample hydrolysis and extraction of total material soluble in lipid solvents; dietary fiber (g) represents the sum of soluble and insoluble dietary fiber determined by an enzymatic method; protein (g) is derived from the total nitrogen content in the food using a food-specific conversion factor (e.g., 6.25); carbohydrate (g) represents the total amount of carbohydrate calculated “by difference” (e.g., calculated per 100 g as the difference between 100 and the sum of the proportions of water, protein, total fat, ash, and alcohol); sodium (mg) is determined by atomic absorption spectrometry. Energy (kcal per 100 g) is calculated as the sum of multiplying the proximate amount by the general Atwater factors (4 kcal/g protein, 4 kcal/g total carbohydrate, 9 kcal/g total fat). Salt (g) is calculated from sodium content converted to grams by a factor of 2.54 [29]. The total amount of nutrients considered was calculated by entering the mass of food offered or consumed.

Dietary records: At the end of the trial, all data from the 7-day dietary record of food consumed outside the kindergarten and the kindergarten 5-day dietary record were entered into the OPEN database [29]. Dietary records were reviewed for any inconsistencies or incomplete information. In instances where such issues arose, a dietitian obtained the necessary data by phone from parents, teachers, or cooks within one week following the completion of the study. Final data on the amount of the seven food groups consumed by each participant were estimated using OPEN and manually for whole grains by a dietitian. OPEN was also used to estimate energy and macronutrient intake data (total fat, protein, carbohydrate, dietary fiber, and sodium).

### 2.7. Statistical Methods

A comprehensive statistical analysis was conducted to evaluate differences in food group intakes between the PG and the CG. The average daily food group intakes during the week (outside and inside the kindergarten) were compared to the average daily food group intakes during the weekend (outside of kindergarten). The total food group intakes per weekday and weekend were also compared to the RDFI. The same comparisons were made separately for the PG and the CG. In addition, a comparison of the quantities of food groups served in kindergartens between the PG and CG was made regarding the amount of leftovers (the difference between food served and consumed).

The Welch two-sample *t*-test was used to compare variables between the PG and CG, whereas a paired sample *t*-test was used to compare variables between weekdays and weekend within each group. A visual inspection of histograms was performed to evaluate the data’s normal distribution. As the assumption of approximate normal distribution remained reasonably intact, the Welch *t*-test, which is well suited for comparing means in instances of non-normal distribution, where variances differ, and in the case of relatively small sample sizes, was used [32]. Analyses were performed separately for all the food groups. To account for multiple comparisons, the Bonferroni correction was employed. All analyses were performed using R version 4.0.2 [33] at a statistical significance level of *p* < 0.05.

## 3. Results

### 3.1. Participants and Settings

**Figure 1:**
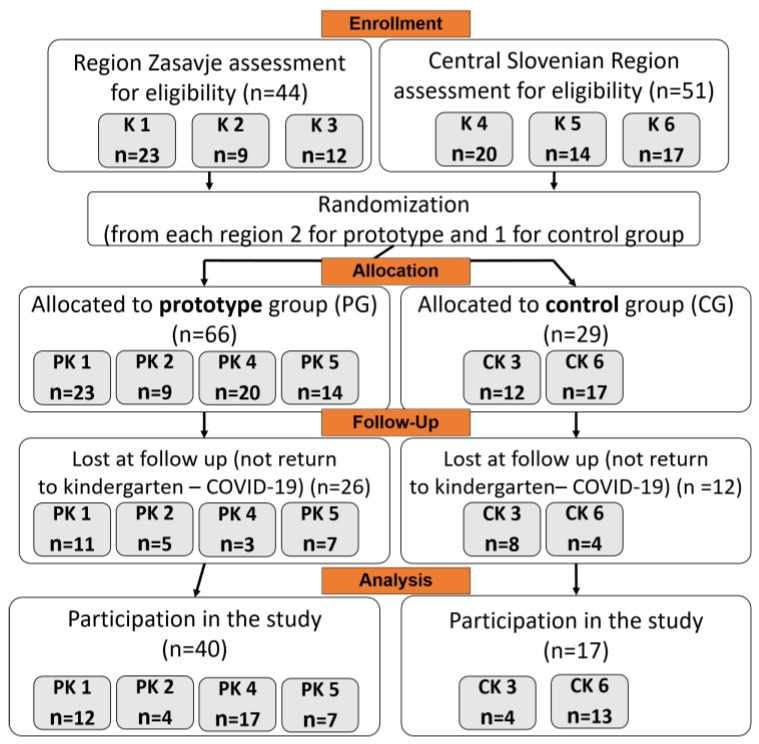
Flowchart showing the enrollment process of participants, assignment to the two study groups, losses to follow-up, and the number of participants completing the study. Note: K, kindergarten; PK, prototype kindergarten; CK, control kindergarten; *n*, number of participants.

Out of the 95 informed consents received before the COVID-19 pandemic, 57 healthy participants completed the study. The dropout rate among participants who began the study in March 2020, before the outbreak of the COVID-19 pandemic, was 15%. In contrast, the dropout rate was higher (47%) among those who began the study in May 2020, after the kindergartens reopened, primarily because participants did not return to the kindergartens. Participants included 5–6-year-old Caucasian children (36 boys, 21 girls). Once enrolled, the children were randomized into the PG and the CG (Figure 1).

### 3.2. Anthropometric Measurements

The mean values within the PG and CG groups were as follows: body height of 118.1 ± 4.7 cm compared to 117.8 ± 3.4 cm, body weight of 22.2 ± 3.6 kg compared to 21.5 ± 3.7 kg, and a BMI of 15.9 ± 1.8 compared to 16.4 ± 2.6 kg/m^2^. No significant differences were observed in the measured anthropometric parameters between the children in either group. Therefore, it can be concluded that the children in the two study groups have comparable quantitative dietary intake requirements.

### 3.3. Food Groups Offered in Kindergartens

Thirty daily kindergarten meals were analyzed; twenty followed the prototype 5-day meal plan, and ten kindergartens the regular 5-day meal plan (Tables S1–S3). All individual foods and composite dishes, such as risotto, stew, pasta, and soup, were classified into seven distinct food groups (Table S4). For the PG meals, the proportion of vegetables ranged from 7–74%, with the most extensive range recorded for tuna in tomato sauce (7–49%), followed by vegetable soup (13–45%). In the CG, the proportion of vegetables in composite dishes ranged from 12–70%. In addition, variations in the amounts of food groups used in the same dishes were also observed in most other composite dishes of the PG meals.

**Figure 2:**
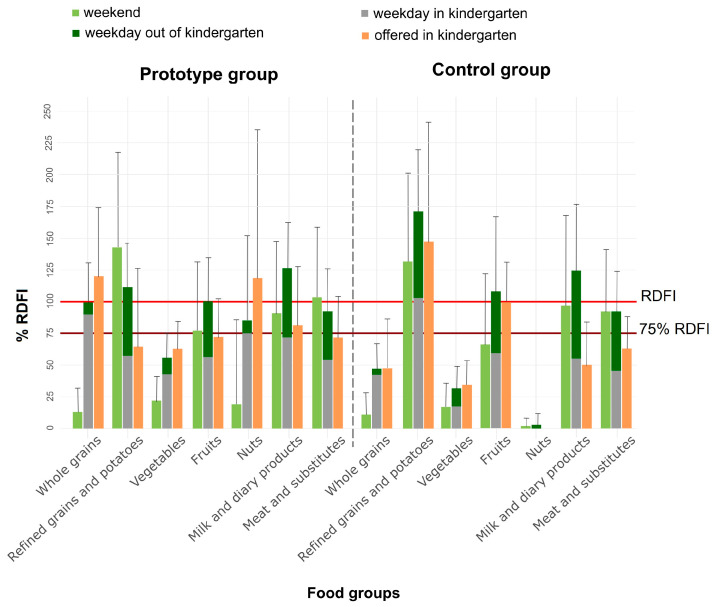
Histogram showing the average amount of food groups in the prototype 5-day and regular kindergarten daily meals (breakfast, lunch, two snacks) compared to RDFI (orange line) and 75% RDFI (red line). Also shown are the contribution of the kindergarten’s daily diet (breakfast, lunch, two snacks) and the outside diet to the daily intake of food groups by the PG participants (*n* = 40) and the CG participants (*n* = 17), compared to RDFI (orange line) and 75% RDFI (red line). Note: RDFI = recommended daily food intake as set out in the dietary Guidelines for healthy eating in educational institutions [13].

The meals in the PG align more with the recommended daily food offer (RDFO) than those in the CG (Figure 2).

Significantly higher average daily amounts of vegetables (188 vs. 103 g), whole grains (137 vs. 54 g), and nuts (14 vs. 0 g) were offered in the PG compared to the CG, while higher amounts of refined foods and potatoes (74 vs. 169 g) and fruits (143 vs. 198 g) were offered in the CG. While the PG meals, on average, contained an adequate daily quantity of nuts (14 g), the CG meals did not include nuts. The availability of milk and dairy products and meat and meat substitutes were similar in both groups. All quantitative offerings of food groups in the PG and CG compared to the guideline recommendations are shown in Table 1.

### 3.4. Energy, Macronutrient, and Sodium Content in Kindergarten Meals

Table 2 shows that prototype kindergarten meals had a significantly higher and more adequate energy, total fat, and dietary fiber content than regular kindergarten meals, while there was no significant difference between the protein and carbohydrate content. Considering the reference values set in the Guidelines [13] for energy and nutrient intake, kindergarten meals provided the participants from the PG with an average of 72% compared with 57% for those in the CG of the Dietary Reference Intake (DRI) for energy. The PG meals contained adequate total fat (72% of the mean DRI value), while the CG meals did not meet the minimum DRI value (64%). The protein content was adequate in all the meals, while the carbohydrate content was low in the PG meals (74% DRI) and even lower in the CG meals (66% DRI). The dietary fiber content was adequate in all meals; however, the PG meals contained significantly more fiber. The analysis also showed that the PG meals contained 5 g of salt and the CG meals contained 4 g of salt, which was above the limit for the maximum allowable daily intake for all meals.

The levels of certain vitamins and minerals associated with the antioxidant activity of foods determined in the PG and CG kindergarten meals are shown in supplementary Table S5. The PG kindergarten meals had significantly higher levels of vitamin E, vitamin C, and zinc than the CG kindergarten meals. The average contents of potassium, iron, zinc, copper, magnesium, manganese, selenium, chromium, and vitamin C were consistent with the DRI in the kindergarten meals of both study groups. Adequate levels of vitamin E and iodine were present only in the PG kindergarten meals, while the levels of vitamin A and calcium were not consistent with the DRI in either group of kindergarten meals.

### 3.5. Food Intake in Kindergartens and Outside Kindergartens on Weekdays by the Prototype and Control Group Participants

The results show (Table 1) that only the kindergarten diet contributed to the significant differences in the total weekday intake of different food groups. Intake outside of kindergartens did not differ significantly among the PG and CG participants.

Comparison between the intake of different food groups inside and outside of kindergarten (Figure 2) shows that in kindergartens, the PG participants, on average, consumed more vegetables, whole grains, and nuts than the CG participants. Compared with the PG participants, the CG participants consumed significantly higher amounts of refined foods and potatoes (57% vs. 103% of the RDFI) and fruit (56% vs. 67% RDFI) in the kindergarten. However, there were no significant differences between the PG and the CG participants regarding the intake of milk and dairy products or meat and substitutes in kindergartens, although the daily intake was higher among the PG participants (72% vs. 55% and 54% vs. 45% of the RDFI). Also, no significant differences existed in the amount of leftovers between the PG and CG participants. The average daily total amount of leftovers in kindergartens (breakfast, lunch, two snacks) for all meals combined was 174 g in the PG and 149 g in the CG (Table S6).

### 3.6. Weekend food Intake among Participants in the Prototype and Control Group Compared with Weekday Intake

During the weekend, the intake of all food groups showed no significant difference between the PG and the CG participants. The intake was insufficient for all food groups except for meat and its substitutes and refined foods and potatoes (Table 1). The lowest intake compared to the RDFI was observed for vegetables, whole grains, and nuts in both study groups. Their average daily intake did not even reach 20%. Conversely, the average intake of milk and dairy products and fruit was somewhat more adequate, corresponding to 91% and 77% of the RDFI, respectively, for the PG participants and 97% and 66% of the RDFI, respectively, for the CG participants.

A comparison of weekdays and weekends revealed that the PG participants consumed significantly higher and thus more adequate amounts of vegetables, whole grains, and nuts during the week, mainly due to the kindergarten meals. There were no significant differences between the intakes of the other food groups.

While the CG participants exhibited a relatively low total intake of whole grain products during the week, it was still significantly higher than that observed during the weekend (43% vs. 11% of RDFI), again with the kindergarten meals accounting for the difference. The average intakes of all other food groups did not differ significantly.

## 4. Discussion

This cross-sectional intervention study is the first to compare the impact of a prototype and regular 5-day kindergarten meal plan in the same country, in line with national dietary guidelines, on the daily intake of key food groups among 5–6-year-old participants. This study also confirms the hypothesis that meticulously designed kindergarten meal plans can significantly contribute to an adequate daily intake of vegetables, fruits, whole grains, and nuts for the PG participants during weekdays. Furthermore, compared to the nutrition provided outside of kindergartens, kindergarten meals contribute to a more optimal intake of nutritious food groups for both the PG and the CG. Notably, the prototype meal plan’s contribution was more pronounced. The more optimal nutritional value, including higher antioxidant capacity, of prototype kindergarten meals was also confirmed in our previous study [35].

So far, few studies have explored the daily intake of food groups and nutrients in preschool children (≤6 years of age). Among these, several examine the contribution of daily nutrition inside and outside of kindergartens [18,19,36], whereas others focus on specific meals, whether at home, in kindergartens, or both settings [25,37,38,39]. However, they all report similar findings, namely that organized kindergarten nutrition is, on average, of higher quality than nutrition outside of kindergartens, a finding confirmed by this study.

Since the primary goal of our study was to investigate if a meticulously designed kindergarten meal plan can effectively contribute to enhancing the daily intake of healthy foods among children, we focused mainly on the offer and intake of food groups in kindergartens. All meal plans in the PG and the CG kindergartens were planned according to the Guidelines [13], which was reflected in the similar offer of all main food groups (milk and dairy product, meat and substitutes), including a total offer of combined fruit and vegetable groups (331 g vs. 301 g) and combined whole grains and refined grains plus potatoes (211 vs. 223 g).

Significant differences existed in the proportion of offered vegetables and fruits, whole grains and refined grains with potatoes, and nuts between the PG and the CG meals (Table 1). These differences resulted mainly from the greater diversity of foods offered. Specifically, the PG meal plan offered on average 14 dishes per day, while the meal plan in the CG comprised an average of 10 dishes per day. The more diverse offer in the PG contributed to a notably higher intake of vegetables, whole grains, and nuts among the PG kindergarten participants. This finding aligns with a recent meta-analysis that variety enhances food intake [40]. Similarly, this study supports Roe et al.’s [41] findings that children consume more vegetables when offered a variety of familiar types of vegetables compared to a single vegetable snack.

Recent research on portion sizes of vegetables and fruits found that larger servings increase children’s daily consumption of these foods; however, larger portions also led to more waste [42]. The current study did not confirm this finding as the amount of leftovers in the PG kindergartens was not significantly higher for any food groups than in the CG kindergartens (Table S6), as the prototype meal plan comprised vegetable and whole grain dishes that children were familiar with. Similarly, Spill et al. [43] have shown the effectiveness of incorporating manipulated vegetables into various well-accepted main dishes to increase vegetable intake [43].

Considering that children are expected to consume more than 60% of their daily energy and nutritional needs (in Slovenia and some other countries, even up to 75%) in kindergartens, the prototype meal plan satisfied the RDFI requirements concerning almost all parameters discussed, unlike the regular meal plan. In addition to that, the 7-day participants’ dietary record showed that the diet outside the kindergarten contained meagre amounts of vegetables, whole grains, and nuts, agreeing with the EFSA that the intake of healthy food groups is inadequate [12]. By comparing the average amounts of food groups consumed during the week and the weekend, when only parents care for children’s nutrition, we can clearly distinguish the positive contribution of kindergarten nutrition, especially in providing an adequate amount of vegetables, fruits, whole grains, and nuts. This effect was also evident among the CG participants, although their weekday nutrition was less optimal than the PG. Namely, during weekdays, participants in both groups consumed the recommended fruit intake, while this was not the case during the weekend, likely reflecting poorer eating habits among Slovenes [16]. The weekday intake of vegetables was not adequate even for the PG participants; however, their average daily weekday intake was significantly more optimal than the average weekend and weekday intake for the CG who were offered too low an amount of vegetables in the kindergarten. The low amount of vegetables in kindergarten meals (approx. 20% of the RDFI) is similarly documented in research from Finland [19] and the US [18,42]. The prototype kindergarten meal plan also supported an adequate daily intake of whole grains on weekdays for the PG participants. For the CG, the whole grain intake was lower during weekdays but significantly higher than at weekends. This finding underscores the need for increased whole grains in kindergarten meal plans, as replacing refined grains with whole grains can help meet the RDFI, especially given the limited intake outside the kindergarten.

As a result of the significantly higher amount of offered vegetables, whole grains, and nuts in the PG meals, it is necessary to highlight the significantly higher content of dietary fiber. A sufficiently high intake of dietary fiber is associated with improved glucose tolerance and lower cardiovascular risk in children [44]. A higher supply of the aforementioned food groups contributed to significantly higher intakes of vitamin C, vitamin E, manganese, and zinc on an average weekday in the PG participants compared to the CG children (Table S5).

This study also investigated the inclusion and consumption of nuts, a subject that had not been previously addressed in the existing literature. The EFSA has noted that European children, on average, have low or negligible nut consumption [12]. However, this study shows how nuts can contribute to achieving an adequate daily intake solely through kindergarten nutrition. In addition to their broad health benefits [22,45], nuts also improve overall diet quality [46], warranting their consideration in dietary guidelines. Caution is essential, however, when providing nuts to children in kindergartens due to choking risks, something that should be considered during preparation and serving. In addition, tree nut allergies are among the eight most common food allergies that may cause severe reactions [47]. Despite all the risks, the benefits of eating nuts may exceed the possible disadvantages; therefore, nuts could be included in kindergarten meal plans, providing that all safety precautions are maintained.

The results of this study coincide with the findings of several other studies [17,18,25], showing that kindergarten nutrition contributes an adequate daily intake of vegetables, fruits, and whole grains. Nevertheless, meticulous adherence to dietary guidelines while designing a kindergarten meal plan can result in an enhanced daily intake of vegetables, fruit, whole grains, and nuts compared to a simplified meal plan design. In addition, special attention must be paid to the use of salt. The salt content in the PG and CG kindergarten meals, as determined by both OPEN and chemical analysis, exceeded the RDI by up to five times (Table 2, [35]). Such an observation should encourage more effective monitoring of the nutritional guideline implementation in kindergartens.

Based on these findings, it is recommended to design kindergarten meals in strict adherence to the guidelines without simplification. While children may not readily embrace certain healthy foods, it is recommended to explore various strategies to enhance their consumption rather than discontinuing their offering [48].

The main strength of this study lies in its comparison of differently designed kindergarten meal plans, all adhering to the same dietary guidelines within the same country. Additionally, a 7-day dietary record, including the amount of food consumed and wasted and covering the weekend, provides a robust insight into the food group intakes in 5–6-year-old children. Precise data on individual food group content were attained by meticulously disaggregating mixed dishes according to weighted recipes. Also, the relatively small number of participants allowed for daily interaction with the children’s parents, teachers, and cooks, preventing errors in filling in the dietary records. The presentation of the data in grams allows for an accurate comparison with other studies. Given the absence of national data on daily food group intake for 5–6-year-olds, the results address this gap, even if they are not representative. Furthermore, the 5-day prototype meal plan is a valuable resource for designing new meal plans and can be readily adapted for other kindergarten settings. This study demonstrates that altering the ratio between vegetables and fruit in favor of vegetables does not lead to significantly increased vegetable leftovers. Nevertheless, it is important to present vegetables in an appealing manner (e.g., small, attractively shaped pieces, attractive colors), opt for vegetables familiar to children, and gradually introduce new varieties.

The findings of this study need to be interpreted in light of its limitations. This study faced challenges in participant recruitment, and higher than expected drop-out, particularly related to parental disinterest and the unique circumstances of the COVID-19 pandemic. As the impact of the pandemic could be considered random, and the government reacted uniformly to it, the results remain relevant for the whole population of kindergarten children in Slovenia. The 7-day dietary records of the study participants were consistent with the results of a recent national food survey conducted in 2018 as part of the EU Menu Study [16], demonstrating that their dietary patterns were not affected by the COVID-19 pandemic. Secondly, the menus in Slovenian kindergartens are designed and planned according to the Guidelines, so the meal plans were not changed despite the occurrence of COVID-19. In addition, this study was conducted at times when the kindergartens were fully operational. Another limitation is that the OPEN tool does not provide discrimination between whole grain and refined starchy food products, and the classification needed to be performed manually. This action could lead to a difference between this study and the results of other studies, as the methodology for calculating whole grain foods is not yet unified. Despite this, this study reveals significant differences between the prototype’s meal plan and food group intake in the prototype and the control group. However, further research with a larger sample is needed to confirm the generalizability of our results.

This study shows how a well-planned and meticulously executed meal plan in a kindergarten can drive positive changes in the nutrition of 5–6-year-old children. Despite the limited number of participants, this study’s findings may be relevant to a broader population of 5–6-year-old children in countries where kindergarten nutrition is well established. These findings could stimulate further research in kindergarten nutrition and prompt educational institutions to reevaluate and adjust their meal plans, potentially leading to healthier and more balanced menus. Herewith, it is worth noting that nutrition in kindergartens depends on several factors. One of the most important is the capacity and level of training of nutritionists and cooks who are empowered to design meal plans and prepare healthy meals according to healthy eating guidelines. Therefore, nationally coordinated strategies and measures are needed to achieve these goals.

## 5. Conclusions

This study highlights the effectiveness of a carefully designed and meticulously executed kindergarten meal plan in increasing the daily intake of vegetables, whole grains, and nuts in 5–6-year-old children. In addition, this study points to the participants’ excessive salt consumption, mainly due to its overuse in kindergartens and household kitchens. The findings underscore the need for additional strategies to promote consistent implementation of dietary guidelines in meal planning and preparation in kindergartens. Because the intake of healthy food groups is inadequate among children worldwide, these findings provide an example of how to improve the daily nutrition of children in educational settings with regulated nutrition.

## Figures and Tables

**Table 1:** Five-day averages of the food groups present in the PG (*n* = 4) and the CG (*n* = 2) and 5/7-day intake of food groups among 5–6-year-olds (*n* = 57) in the PG (*n* = 40) and CG (*n* = 17), according to meal location. The data were obtained from the 7-day dietary records for inside and outside the kindergartens.

	Food Groups ^1^	Meat and Substitutes (g)	Milk and Dairy Products (g)	Fruits (g)	Vegetables (g)	Nuts (g)	Whole Grains (g)	Refined Grains and Potatoes (g)
Variable of interest	RDFI ^2^ 4–6 years (75% RDFI) ^3^	110 (82.5)	400 ^4^ (300)	200 (150)	300 (225)	11 ^5^ (8)	115 ^6^ (86)	115 ^6^ (86)
Offered in kindergarten Mean (SD) ^7^	PG	78 (36)	325 (162)	143 (61)	188 (66)	14 (13)	137 (62)	74 (71)
	CG	69 (28)	200 (136)	198 (64)	103 (58)	0	54 (45)	169 (108)
*p*-value		1	0.231	0.233	0.011 *	<0.001 *	0.002 *	0.152
Weekday in kindergartens Mean (SD) ^8^	PG	59 (23)	289 (94)	112 (34)	136 (48)	8 (6)	106 (35)	66 (23)
	CG	50 (24)	220 (101)	134 (74)	60 (51)	0	49 (26)	119 (48)
*p*-value		1	0.137	1	<0.001 *	<0.001 *	<0.001 *	0.002 *
Weekdays outside kindergartens Mean (SD) ^9^	PG	42 (28)	216 (111)	88 (65)	33 (31)	0.8 (3)	9 (13)	62 (39)
	CG	51 (29)	275 (199)	82 (64)	35 (36)	0.3 (1)	5 (10)	78 (39)
*p*-value		1	1	1	1	1	1	1
Total weekdays Mean (SD)	PG	101 (37)	505 (145)	200 (80)	169 (59)	8.3 (7)	115 (36)	128 (40)
	CG	101 (35)	495 (210)	216 (118)	95 (53)	0.3 (1)	54 (23)	197 (56)
*p*-value		1	1	1	<0.001 *	<0.001 *	<0.001 *	0.005 *
Total Weekend Mean (SD) ^10^	PG	113 (61)	365 (227)	153 (109)	66 (58)	2 (7)	13 (22)	164 (86)
	CG	101 (54)	388 (285)	131 (112)	52 (57)	0.2 (0.7)	13 (20)	152 (80)
*p*-value		1	1	1	1	0.288	1	1

PG, prototype group; CG, control group; SD, standard deviation; RDFI, recommended daily food intake; RDFO, recommended daily food offer. ^1^ The food groups represent the groups used in the dietary assessment tool OPEN; starchy foods were separated into whole grains and refined grains, and potatoes; ^2^ RDFI for 4–6-year-old children set in Guidelines [13]; ^3^ 75% RDFI/O recommended food intake/offer in kindergarten; ^4^ RDFI for milk and dairy products is two units, of which one unit represents 200 mL of milk or yogurt or 30 g of soft cheese (cottage cheese, cheese spread) or 15 g of hard cheese (e.g., parmesan, edam, and gouda) [13]. Based on the recipes, milk and dairy units were converted to g as follows: milk and yogurt as actual intake, soft cheese = (amount × 200)/30 hard cheese = (amount × 200)/15; ^5^ RDFI was calculated based on the doses recommended for adults, adjusted to children’s body weight (on average 0.43 g/kg of body weight [34]; ^6^ RDFI for starchy foods is nine units, of which one unit represents 20–30 g of starchy foods; at least half of them should be whole grain [13]; ^7^ Mean (SD) are the mean values in the PG and the CG meals, based on weighed recipes and analyzed with OPKP; ^8^ Mean (SD) are the mean values of 5 consecutive days’ (without weekends) intake of food groups in the kindergartens for the PG participants (*n* = 40) and the CG participants (*n* = 17), obtained from 5-day kindergarten weighed dietary record completed by teachers; ^9^ Mean (SD) are the mean values of 5-weekday (without weekend) intake of food groups outside the kindergarten for the PG participants (*n* = 40) and the CG participants (*n* = 17), obtained from 7-day weighed or estimated dietary record, completed by parents; ^10^ Mean (SD) are the mean values of weekend (2-day) intake of food groups by the PG participants (*n* = 40) and the CG participants (*n* = 17), obtained from 7-day weighed or estimated dietary record, completed by participants’ parents; * values of average offer/intake amounts in the same column are significantly different between the PG and the CG (*p* < 0.05).

**Table 2:** An average 5-day energy content and approximate composition of meals prepared by kindergartens within the prototype (*n* = 4) and regular (*n* = 2) meal plan and average 5/7-day energy content and approximate composition of meals, consumed according to eating locations, separately for PG participants (*n* = 40) and CG participants (*n* = 17).

		Energy (kcal)	Total Fat (g)	Protein (g)	CH (g)	DF (g)	Na (mg)
Variable of interest	DRI ^1^ 4–6 years. (75% DRI) ^2^	1550 (1162)	52–60 (39–45)	39–58 (29–44)	>194 (>146)	˃15 (˃11)	<1180 (880)
Offered in kindergartens Mean (SD) ^3^	PG	1115 (221)	43 (12)	39 (7)	143 (31)	20 (5)	1983 (1148)
	CG	883 (178)	25 (12)	35 (8)	128 (30)	14 (5)	1605 (718)
*p*-value		0.031 *	<0.001 *	1	1	0.017 *	1
Weekday in kindergartens Mean (SD) ^4^	PG	830 (176)	30 (8)	29 (6)	112 (23)	15 (3)	1478 (400)
	CG	664 (239)	20 (8)	28 (11)	94 (34)	10 (4)	1126 (521)
*p*-value		0.097	<0.001 *	1	0.334	<0.001 *	0.196
Weekdays outside kindergartens Mean (SD) ^5^	PG	660 (206)	25 (9)	22 (8)	87 (30)	6 (2)	687 (286)
	CG	799 (289)	32 (15)	27 (8)	101 (37)	6 (3)	757 (366)
*p*-value		0.508	0.587	0.227	0.956	1	1
Total weekdays Mean (SD)	PG	1486 (250)	55 (12)	50 (10)	198 (35)	21 (4)	2154 (463)
	CG	1462 (317)	52 (16)	54 (11)	195 (46)	16 (5)	1910 (517)
*p*-value		1	1	1	1	0.010 *	0.627
Total Weekend Mean (SD) ^6^	PG	1336 (374)	50 (17)	48 (14)	173 (56)	11 (5)	1648 (852)
	CG	1440 (289)	57 (15)	48 (8)	183 (44)	12 (3)	1519 (527)
*p*-value		1	0.808	1	1	1	1

PG, prototype group; CG, control group; SD, standard deviation; CH, carbohydrates; DF, dietary fiber; Na, sodium. ^1^ DRI—Dietary Reference Intake per day for 4–6-year-old children with PAL (physical activity level) 1.6 (moderately active) [13]; ^2^ 75%—DRI for time spent in kindergarten; ^3^ Mean (SD) mean values of offered PG and CG kindergartens meals, based on weighed recipes and analyzed with OPKP; ^4^ Mean (SD) are the mean values of 5-weekday (without weekend) intake and energy, macronutrient, and sodium consumption in kindergartens by PG and CG participants; ^5^ Mean (SD) mean values of 5-weekday (without weekends) intake and energy, macronutrient, and sodium consumption outside kindergartens by PG and CG participants; ^6^ Mean (SD) mean values of weekend (2-day) intake and energy, macronutrient, and sodium consumption by PG and CG participants; * values in the same column of average offer/intake amounts are significantly different between PG and CG participants (*p* < 0.05).

## Data Availability

The data presented in this study are available on request from the corresponding author.

## Supplementary Materials

The following supporting information can be downloaded at https://www.mdpi.com/article/10.3390/nu15184088/s1, Table S1: prototype 5-day kindergarten meal plan, including the recommended serving portions (g/child) and approximate energy intakes by individual meals; Table S2: regular 5-day kindergarten meal plan from Zasavje region including the recommended serving portions (g/child) and approximate energy intakes by individual meals; Table S3: regular 5-day kindergarten meal plan from Central Slovenia region including the recommended serving portions (g/child) and approximate energy intakes by individual meals; Table S4: classification of foods and mixed dishes from 5-day kindergarten meal plans into food groups; Table S5: an average 5-day vitamin and mineral content of meals prepared by kindergartens within the prototype (*n* = 4) and regular (*n* = 2) meal plan and average 5/7-day vitamin and mineral content of meals, consumed according to eating locations, separately for PG participants (*n* = 40) and CG participants (*n* = 17); Table S6: leftovers in PG kindergartens and CG kindergartens.

## Abbreviations

NCDs—noncommunicable diseases; EFSA—European Food Safety Authority; Guidelines—Guidelines for Healthy Eating in Educational Institutions; BMI—body mass index; OPEN—Open Platform for Clinical Nutrition; PG—prototype group; CG—control group; SD—standard deviation; DRI—Dietary Reference Intake; RDFI—recommended daily food intake; RDFO—recommended daily food offer; K—kindergarten; PK—prototype kindergarten; CK—control kindergarten; Na—sodium; DF—dietary fiber; CH—carbohydrates.
