# Supplementary material for: Can Kindergarten Meals Improve the Daily Intake of Vegetables, Whole Grains, and Nuts among Preschool Children? A Randomized Controlled Evaluation
Source: Nutrients. 2023 Sep 21;15(18):4088. doi: 10.3390/nu15184088 (PMC10536440; doi:10.3390/nu15184088)
Supplement: Supplementary file 1 [file nutrients-15-04088-s001.zip › nutrients-2533071-supplementary.pdf]

Online Supplementary materials

Online Supplementary materials ……………………………………………………………………………….…..…1

Table S1 Prototype 5-day kindergarten meal plan, including the recommended serving portions (g/child) and approximate energy intakes by individual meals………………………………………………………………………..…2

Table S2 Regular 5-day kindergarten meal plan from Zasavje region including the recommended serving portions (g/child) and approximate energy intakes by individual meals…………………………………………………….…3

Table S3 Regular 5-day kindergarten meal plan from Central Slovenia region including the recommended serving portions (g/child) and approximate energy intakes by individual meals……………………………………………..4

Table S4 Classification of foods and mixed dishes from 5-day kindergarten meal plans into food groups………...………5

Table S5: An average 5-day vitamin in mineral content of meals prepared by kinder-gartens within the prototype (n=4) and regular (n=2) meal plan and average 5/7-day vitamin and mineral content of meals, consumed according to eating locations, separately for PG participants (n=40) and CG participants (n=17)…………………….7

Table S6: Leftovers in PG kindergartens and CG kindergartens………………………………………………………….9

References……………………………………………………..……………………………………………………...…10

Table S1 Prototype 5-day kindergarten meal plan, including the recommended serving portions (g/child) and approximate energy intakes by individual meals.

	Breakfast 20%*	Morning snack 5%*	Lunch 30-35%*	Afternoon snack 15%*
1st DAY Thursday	hot cocoa (180 g), wheat germ bread (60 g), cottage spread with chives (30 g), slice of apple (20 g)	cherry tomatoes (50 g), radish (50 g)	beef soup (170 g) with noodles (50 g), beef (60 g), sauté potatoes (130 g), steamed broccoli (25 g) and cauliflower (25 g), red cabbage salad (50 g)	blueberries (100 g), buckwheat bread with walnuts (40 g)
2nd DAY Friday	herbal tea with honey and lemon (150 g), spelt bread (70 g), egg spread with vegetable and chicken breast salami (30 g), orange pepper (50 g), slice of apple (20 g)	kiwi (50 g), raspberry (50 g)	kale stew with chicken and quinoa (260 g), rye bread (30 g), yogurt panna cotta (80 g) with blueberry sauce (50 g), 100% orange juice (120 g)	spelt bread roll (50 g), red pepper (50 g), black olives (6 g), cheese (20 g)
3rd DAY Monday	millet porridge with milk (280 g), cinnamon and ground chocolate topping (3 g), slice of apple (20 g)	dried cranberries (15 g) and plums (25 g), walnuts (15 g)	broccoli soup (188 g), whole grain spaghetti (130 g), tuna in tomato sauce with pine nuts and basil (147 g), green salad (50 g) with pomegranate (5 g)	apple sauce with cinnamon and cloves (130 g), whole grain breadsticks (grissini) (20 g)
4th DAY Tuesday	peppermint tea with honey (150 g), sesame bread roll (70 g), chicken breast salami (15 g), edam cheese (15 g), tomato (50 g), slice of apple (20 g)	strawberries (100 g)	barley stew with turkey (260 g), rye bread (20 g), buckwheat cubes with dark chocolate and apples (80 g), lemonade (140 g)	yogurt (100 g) with raspberry puree (50 g) and ground hazelnuts (20 g), multigrain bread (20 g)
5th DAY Wednesday	cereal coffee with milk (160 g), whole grain bread roll (60 g), dried cranberries (30 g), walnuts (30 g), slice of apple (20 g)	blueberries (100 g)	tomato soup (199 g) with noodles (49 g), baked brown rice with chicken and vegetables (203 g), parmesan cheese (5 g), beetroot salad (59 g)	nuts bar (42 g), 100% pomegranate juice (150 g)

* Distribution of recommended energy intake throughout the day by individual meals [1]

Table S2 Regular 5-day kindergarten meal plan from Zasavje region including the recommended serving portions (g/child) and approximate energy intakes by individual meals.

	**Breakfast 20%***	**Morning snack 5%***	**Lunch 30-35%***	**Afternoon snack 15%***
1st DAY Thursday	fruit tea with sugar (190 g), apricot jam croissant (85 g)	banana (50 g), pear (45 g)	salmon fillet (70 g), with sauce of garlic, parsley and olive oil (3g), boiled potatoes (200 g), beetroot (90 g)	banana (130 g), buckwheat bread (70 g)
2nd DAY Friday	tea (120 g), eco white bread (30 g), eco veal hot dog (50 g), mustard (10 g)	banana (60 g), pear (96 g)	chicken ragout (120 g), wide white noodles (200 g), green lettuce (20 g)	eco black bread (30 g), apple (120 g)
3rd DAY Monday	tea (120 g), semi white bread (50 g), cooked prosciutto (30 g), pickle (10 g)	apple (40 g), pear (60 g)	beef steak (50 g) in carrot sauce (110 g) roasted potatoes (290 g), green salad (40 g)	banana (140 g), whole grain bread (30 g)
4th DAY Tuesday	cocoa (120 g), cheese bread roll (80 g)	apple (50 g), pear (50 g)	bean stew with white pasta (360 g), marble cake (50 g)	melon (100 g), whole grain cookies (20 g)
5th DAY Wednesday	white wheat grits in milk (190 g), chocolate topping (1 g)	apple (50 g), pear (50 g), banana (60 g)	risotto (rice, barley, spelt) with turkey and vegetables (260 g), green salad with boiled egg (60 g)	pear (120 g), breadsticks (grissini) (20 g)

* Distribution of recommended energy intake throughout the day by individual meals [1]

Table S3 Regular 5-day kindergarten meal plan from Central Slovenia region including the recommended serving portions (g/child) and approximate energy intakes by individual meals.

	Breakfast 20%*	Morning snack 5%*	Lunch 30-35%*	Afternoon snack 15%*
1st DAY Thursday	eco milk (120 g), black bread (70 g), honey and butter spread (20 g)	apple (80 g)	beef and vegetable lasagne (280 g), green salad with chickpeas (35 g)	banana (120 g)
2nd DAY Friday	tea (180 g), eco spelt bread (65 g), turkey salami (30 g), sour red pepper (15 g)	nectarine (80 g)	barley stew (280 g), cottage cheese strudel (110 g)	eco spelt bread (30 g), apple (90 g)
3rd DAY Monday	white coffee (140 g), semi white bread (65 g), butter (15 g), apricot jam (20 g)	nectarine (80 g)	roasted chicken legs (45g), mashed potatoes (200 g), green salad with carrots (30 g)	rice waffle (10 g), apple (80 g)
4th DAY Tuesday	millet porridge with milk (240 g), chocolate topping (3 g)	banana (70 g), eco carrot (10 g)	beef goulash (140 g), steamed bread dumplings (60 g), green salad with corn (30 g)	pear (80 g), whole grain toast (10 g)
5th DAY Wednesday	tea (300 g) eco whole grain bread roll (70 g), vegetable spread (20 g)	apricot (80 g)	carrot soup (140 g), fried hake (80 g), salad with cabbage and potatoes (150 g)	vanilla yogurt (155 g), eco whole grain bread (20 g)

* Distribution of recommended energy intake throughout the day by individual meals [1]

Table S4 Classification of foods and mixed dishes from 5-day kindergarten meal plans into food groups

Food group *	Served food	
	Prototype group [1]	Control group
Meat and substitutes (salami, eggs)	[2] meat and vegetable spread (chicken salami) (41-80%), [2] barley stew (18-35%), [2] buckwheat cubes (eggs) (10-30%), [2] baked brown rice with chicken and vegetables (23-30%), [2] tuna with tomato sauce (44-93%), [2] kale stew with chicken and quinoa (16-33%), Beef, fish, poultry, tuna, eggs, chicken breast salami	[2] beef steak in carrot sauce (61%), [2] turkey ragu (40%), [2] bean stew with white pasta (10%), [2] marble cake (eggs) (11%), [2] lasagne (17%), [2] barley stew (smoked pork) (6%), [2] cottage cheese strudel (eggs) (12%), [2] beef goulash (26%) salmon, chicken, smoked bacon, veal hot dog, cooked prosciutto, [2] wiener style hake (83%),
Milk and dairy product	whole milk (3,5%), yogurt (3,5%), kefir (3,5%), cottage cheese, cocoa, non-caffein white coffee, cheese, yogurt panna cotta, [2] meat and vegetable spread (cheese) (41-80%), [2] millet porridge with milk (23-90%)	milk (3,5%), cocoa (3,5%), [2] turkey ragu (23%), [2] marble cake (12%), [2] corn grits (89%), [2] risotto with turkey and vegetables (parmesan) (34%), [2] lasagne (47%), *barley stew (6%), [2] cottage cheese strudel (54%), [2] vegetable spread (42%), [2] millet porridge with milk (93,5%), vanilla yogurt
Fruits	blueberries, raspberry puree, strawberries, dried cranberry, apple, kiwi, raspberry, dried plums, blueberry sauce, pomegranate, [2] buckwheat cubes (apples) (0-31%), [2] nuts bar (cranberries, raisins) (17-51%)	banana, pear, apple, melon, nectarine, [2] cottage cheese strudel (apple) (5%)
Vegetables	[2] meat and vegetable spread (19-44%), [2] tomato soup (13-45%), [2] broccoli soup (32-45%), [2] tuna with tomato sauce (7-49%), [2] barley stew (28-37%), [2] baked brown rice with chicken and vegetables (26-57%), [2] kale stew with chicken and quinoa (54-74%), red pepper, olives, cherry tomatoes, turnip, tomato, orange pepper, steamed broccoli and cauliflower, red cabbage salad, red beets, green salad	[2] beef steak in carrot sauce (37%), [2] turkey ragu (12%), [2] bean stew with white pasta (70%), [2] risotto with turkey and vegetables (36%), [2] lasagne (26%), [2] barley stew (27%), [2] beef goulash (31%), 2 vegetable spread (58%), green salad, red beets, pickle, [2] cabbage salad with potatoes (42%), corn, [2] carrot soup (28%),
Nuts	ground hazelnuts, pine nuts, walnuts, [2] buckwheat cubes (walnuts) (0-9%), [2] nuts bar (almonds, walnuts, pumpkin seeds) (16-28%), [2] tuna with tomato sauce (pine nuts) (4-7%)	/
[3] Whole-grain foods	[2] barley stew (7-45%), [2] baked brown rice with chicken and vegetables (20-48%), [2] kale stew with chicken and quinoa (0-2%), Buckwheat bread with walnuts, whole grain spaghetti, multigrain bread, whole grain bread roll, whole spelt bread, [2] buckwheat cubes (13-27%), [2] nuts bar (oatmeal) (7-12%), [2] millet porridge with milk (10-57%), whole grain bread sticks,	[2] marble cake (14%), [2] corn grits (9%), [2] risotto with turkey and vegetables (13%), [2] barley stew (21%), [2] millet porridge with milk (6,5%), whole grain cookies, whole grain toast, whole grain bread, black bread, buckwheat bread,

Food group *	Served food	
	Prototype group [1]	Control group
[3] Refined grain foods and potatoes	[2] barley stew (0-32%), [2] buckwheat cubes (8-28%), [2] kale stew with chicken and quinoa (0-16%), wheat germ bread, sesame bread roll, noodles, potatoes	[2] turkey ragout (6%), [2] bean stew with white pasta (20%), [2] marble cake (14%), [2] risotto with turkey and vegetables (17%), [2] lasagne (10%), [2] barley stew (46%), [2] cottage cheese strudel (21%), [2] Vienna style hake (17%), apricot jam croissant, white bread, white breadsticks, potatoes, white pasta, risotto, cheese bread roll, rice waffle, refined spelt bread, [2] cabbage salad with potatoes (58%), steamed bread dumplings,

* food groups set out in the Guidelines [1] and in dietary assessment tool OPEN [2]

[1] PG kindergartens used the same meal plan, but their own recipes; therefore, the proportion of food groups represented in mixed dishes varies

[2] mixed dishes were classified into food groups manually, according to the weighed recipes. Numbers in parentheses represent a percentage of total weight of mixed dish from a certain food group.

[3] starchy foods were manually divided into whole grain foods and refined grain foods

Table S5 An average 5-day vitamin in mineral content of meals prepared by kindergartens within the prototype (n=4) and regular (n=2) meal plan and average 5/7-day vitamin and mineral content of meals, consumed according to eating locations, separately for PG participants (n=40) and CG participants (n=17)

Variable of interest		Vit A µg RAE[7]	Vit E Mg[8]	Vit C mg	K mg	Ca mg	Mg mg	Fe mg	I µg	Zn mg	Cu µg	Mn µg	Cr µg	Se µg
	DRI[1] 4-6 y (75% DRI)[2] .	700 (525)	8 (6)	30 (23)	1300 (975)	750 (563)	120 (90)	8 (6)	120 (90)	4 (3)	500-100 (375-750)	1500-2000 (1125-1500)	20-80 (15-60)	20 (15)
Offered in kindergartens Mean (SD)[3]	PG	366 (214)	6.2 (2.0)	108 (61)	1831 (482)	468 (103)	322 (190)	9.4 (2.0)	95 (78)	5.8 (1.6)	1081 (289)	3761 (986)	27 (13)	43 (19)
	CG	372 (180)	3.7 (1.9)	41 (21)	1610 (429)	348 (188)	232 (111)	8.2 (2.5)	79 (69)	4.6 (1.9)	919 (474)	2259 (1059)	39 (19)	70 (87)
p-value		1	0.039*	0.002*	1	1	1	1	1	0.019*	1	1	0.174	1
Weekday in kindergartens Mean (SD)[4]	PG	293 (124)	4.5 (1.3)	88 (26)	1426 (321)	393 (103)	257 (76)	7.2 (1.4)	71 (23)	4.5 (0.9)	856 (176)	2841 (681)	22 (5)	32 (10)
	CG	217 (138)	2.7 (1.8)	30 (15)	1216 (468)	367 (204)	176 (81)	5.8 (2.4)	66 (46)	3.7 (1.7)	678 (300)	2080 (1294)	23 (10)	76 (67)
p-value		0.715	0,017*	< 0,001*	1	0.519	1	1	0.383	0.395	1	0.205	0.042*	0.715
Weekdays outside kindergartens Mean (SD)[5]	PG	147 (137)	2.6 (1.4)	33 (20)	830 (328)	309 (124)	97 (38)	6.5 (13)	30 (23)	3.3 (4.6)	408 (166)	1022 (551)	21 (70)	27 (25)
	CG	433 (971)	3.3 (2.2)	38 (25)	959 (306)	415 (184)	103 (39)	5.6 (2.4)	32 (18)	3.0 (1.1)	499 (207)	1083 (532)	8.7 (5.2)	29 (9)
p-value		1	1	1	1	1	1	1	1	1	1	1	1	1
Total weekdays Mean (SD)	PG	426 (191)	6.8 (1.7)	116 (34)	2189 (436)	683 (162)	343 (84)	13.4 (13.7)	98 (35)	7.6 (4.6)	1228 (222)	3729 (720)	42 (71)	58 (27)
	CG	612 (992)	5.5 (2.4)	63 (28)	2017 (410)	727 (210)	254 (67)	10.6 (2.8)	87 (37)	6.3 (1.5)	1082 (267)	2826 (1006)	29 (12)	90 (33)
p-value		1	0.502	< 0,001*	1	1	1	1	0.689	0.033*	1	0.018*	1	0.502

Total Weekend Mean (SD)[6]	PG	393 (381)	5.8 (3.3)	71 (47)	1664 (657)	556 (239)	205 (104)	9.6 (3.4)	57 (31)	5.3 (1.8)	916 (617)	2017 (772)	20 (17)	56 (31)
	CG	298 (156)	5.8 (2.5)	60 (39)	1638 (330)	588 (191)	171 (42)	9.8 (2.5)	66 (38)	4.9 (1.3)	851 (291)	1877 (504)	20 (16)	50 (25)
p-value		1	1	1	1	1	1	1	1	1	1	1	1	1

DRI, – Dietary Reference Intake; RAE, Retinol activity equivalent; PG, prototype group; CG, control group; SD, Standard Deviation; Vit A, Vitamin A; Vit E, Vitamin E; Vit C, Vitamin C; K, Potassium; Ca, Calcium; Mg, Magnesium; Fe, Iron; I, Iodine; Zn, Zinc; Cu, Copper; Mn, Manganese; Cr; Chromium; Se, Selenium

[1]DRI – Dietary Reference Intake per day for 4-6-year-old children with PAL (Physical Activity Level) [13]

[2]75% – DRI for time spent in kindergarten

[3] Mean (SD) Mean values of offered PG and CG kindergartens meals, based on weighed recipes and analysed with OPKP

[4] Mean (SD) are the Mean values of 5-weekday (without weekend) vitamin and mineral intake by PG and CG participants

[5] Mean (SD) Mean values of 5-weekday (without weekends) vitamin and mineral intake by PG and CG participants

[6] Mean (SD) Mean values of weekend (2-day) vitamin and mineral intake by PG and CG participants

[7]DRI for Vitamin A are given as retinol activity equivalents (RAE)

[8]1 mg Vit E (alpha-tocopherol) is equivalent to 1 mg RRR-alpha tocopherol (d-alpha-tocopherol)

*Values in the same column of average offer /intake amounts are significantly different between PG and CG participants ($p<0.05$)

Table S6 Leftovers in PG kindergartens and CG kindergartens

		Meat and substitutes (g)	Milk and dairy products (g)	Fruits (g)	Vegetables (g)	Nuts (g)	Whole grains (g)	Refined grains and potatoes (g)
Leftovers in kindergartens Mean (SD) [1]	PG	20 (21)	17 (82)	33 (41)	50 (45)	5 (6)	31 (33)	18 (17)
	CG	10 (33)	18 (70)	52 (68)	34 (56)	0	12 (25)	23 (74)
p value		1	1	1	1	/	0.146	1

PG, prototype group; CG, control group; SD, standard deviation

[1] Mean (SD) Mean values of daily leftovers in kindergartens for each food group separately, obtained from differences between amount of offered food groups in kindergartens and amount of consumed food groups in PG and CG participants (5-day dietary record completed by participants teachers). In some cases, standard deviation is below zero, because some participants ate more than was offered (because other participants did not)

References

1. Gabrijelčič Blenkuš M, Pograjc L, Gregorčič M, Adamič M, Čampa A. Smernice zdravega prehranjevanja v vzgojno-izobraževalnih ustanovah (od prvega leta starosti naprej). *Ministry of Health* **2005.** Available online: https://nijz.si/wp-content/uploads/2022/07/smernice_zdravega_prehranjevanja_v_viu.pdf (accessed 15 September 2022)
2. Korošec M, Golob T, Bertoncelj J, Stibilj V, Seljak BK (2013) The Slovenian food composition database. *Food Chem* **2013**, 140(3), 495-499. https://doi.org/10.1016/j.foodchem.2013.01.005
